# Beneficial effects of training in self-distancing and perspective broadening for people with a history of recurrent depression

**DOI:** 10.1016/j.brat.2017.05.008

**Published:** 2017-05-11

**Authors:** Emma Travers-Hill, Barnaby D. Dunn, Laura Hoppitt, Caitlin Hitchcock, Tim Dalgleish

**Affiliations:** a Medical Research Council Cognition and Brain Sciences Unit, UK; b University of Exeter, UK; c Medical Research Council Cognition and Brain Sciences Unit, Cambridgeshire and Peterborough NHS Foundation Trust, UK

**Keywords:** Self-distancing, Perspective broadening, Decentering, Depression, Cognitive training

## Abstract

Cognitive training designed to recalibrate maladaptive aspects of cognitive-affective processing associated with the presence of emotional disorder can deliver clinical benefits. This study examined the ability of an integrated training in self-distancing and perspective broadening (SD-PB) with respect to distressing experiences to deliver such benefits in individuals with a history of recurrent depression (≥3 prior episodes), currently in remission. Relative to an overcoming avoidance (OA) control condition, SD-PB: a) reduced distress to upsetting memories and to newly encountered events, both during training when explicitly instructed to apply SD-PB techniques, and after-training in the absence of explicit instructions; b) enhanced capacity to self-distance from and broaden perspectives on participants' experiences; c) reduced residual symptoms of depression. These data provide initial support for SD-PB as a low-intensity cognitive training providing a spectrum of cognitive and affective benefits for those with recurrent depression who are at elevated risk of future episodes.

Major depressive disorder typically runs a relapsing and recurrent course (Judd, 1997). Without ongoing clinical care those with depression have a high risk of repeated depressive relapses throughout their life, even after successful acute treatment (Kupfer, 1991). Cognitive models of depression focus on the idea that established patterns of maladaptive cognitive processing persist during remission from depressive episodes, thus conferring vulnerability to later relapse (Power & Dalgleish, 2015; Teasdale, 1988). If these cognitive factors that make people vulnerable to relapse can be attenuated whilst sufferers are in remission, the relapsing course of depression could potentially be broken or weakened.

A number of psychological interventions have been developed that can be used to target such cognitive change in remitted depressed individuals, most notably cognitive-behavior therapy (CBT; Vittengl, Clark, Dunn, & Jarrett, 2007) and mindfulness-based cognitive therapy (MBCT; Segal, Williams, & Teasdale, 2012). However, these interventions are complex, intensive and require specialized therapist training. Thus, although there is accumulating evidence for the efficacy of these approaches (Hofmann, Asnaani, Vonk, Sawyer, & Fang, 2012; Kuyken et al., 2016), their wide-spread availability is currently limited. There is consequently a strong case for developing lower intensity cognitive interventions which target the same proposed maladaptive vulnerability processes, and can also be delivered during periods of depressive remission when clients are feeling psychologically well. Interventions drawn from basic science that aim to reduce depressogenic processing biases have been effective in reducing these vulnerabilities in depressed samples (e.g., autobiographical memory training, Neshat-Doost et al., 2013; cognitive bias modification, MacLeod & Mathews, 2012), and we aimed to expand upon this work by testing a novel training paradigm specifically designed for individuals remitted from depression.

In this study we evaluate a cognitive training protocol derived from two areas of basic science relevant to depression – self-distancing (Kross & Ayduk, 2011) and perspective broadening (Schartau, Dalgleish, & Dunn, 2009; Trope & Liberman, 2010). The theoretical basis of research in both of these domains is consistent with cognitive models of depression and of depressive relapse (Teasdale, 1988). Both domains focus on cognitive processes that are also the target of existing complex psychological interventions for depression prevention such as CBT and MBCT. Finally, research in both domains focuses on cognitive and affective change across time, as opposed to simply mapping the nature of cognition-emotion interactions, thus providing a platform for cognitive intervention development.

The meta-cognitive process model (Bernstein et al., 2015) defines three separate components of decentering: meta-awareness of subjective experience; reduced reactivity to thought content; and disidentification from internal experience. The self-distancing (SD) element of decentering refers to the process of mentally stepping back from an experience in order to examine it as separate from the self, and from the perspective of a distanced observer to facilitate disidentification from internal experience. Kross, Ayduk and colleagues have shown in a novel series of studies that analyzing the meaning of memories and experiences (e.g. thinking about why they may have occurred) from a self-distanced perspective can reap mental health benefits (see Kross & Ayduk, 2011; for a summary). In their key study looking at depression, Kross, Card, Deldin, Clifton, and Ayduk (2012) found that asking depressed individuals to think about the meaning of a recent upsetting life event from a self-distanced stance, as opposed to from an immersed standpoint, resulted in reduced depressotypic thought and negative affect and an attenuated tendency to focus on emotionally arousing aspects of the experience. These findings suggest that systematic practice in SD to scaffold the reappraisal of difficult material may accrue adaptive benefits in how depressed people process upsetting events in their lives. Indeed, this reappraisal element appears to be critical as there is evidence that self-distancing alone, in the form of simply adopting an observer perspective on mentally simulated events, can be harmful (e.g., Kuyken & Moulds, 2009).^1^

Perspective Broadening (PB) refers to the psychological process of contextualizing experiences within broader mental frameworks-seeing the bigger picture (Schartau et al., 2009; Trope & Liberman, 2010). Perspective can be broadened along different 'dimensions'. For example, PB along the temporal dimension could involve contemplating how you might feel about a recent event in a week's time or a year's time. Perspective can also be broadened by thinking about how a given event (e.g. a bad evening out with a friend) compares to other similar events in the past (other times spent with that friend), how experiences in one life domain (e.g. a relationship) compare to the broader context of other domains (work, friendships, family etc.), how the person might think about the event if it happened to someone else, or how someone else might think about the event if you told them about it. Previous work has shown that a one-off training session that teaches people with sub-clinical levels of depressive affect to broaden their perspective on memories and novel events in these different ways significantly reduces the self-reported and psychophysiological distress they experience in relation to such events (Schartau et al., 2009). This work sits against a wider backdrop of research suggesting that such broader mind-sets are associated with more positive emotional states (e.g., Garland et al., 2010; Watkins, Teasdale, & Williams, 2000) and that psychological treatments that capitalize on these cognitive dynamics are likely to be beneficial (Fredrickson, 2001; Wood & Tarrier, 2010).

In the current study we examined the cognitive and affective benefits of systematically training individuals with a diagnosis of recurrent major depressive disorder, currently in remission, in the use of a psychological technique that combines the core elements of both SD and PB. In doing so we took the basic SD approach as our starting point (Kross et al., 2012) but instead of encouraging participants to only ask ‘why?’ from a distance, we trained them instead to use this distanced mental vantage point to contextualize their experiences within a range of broader perspectives, focusing on the different perspective dimensions outlined above. Our rationale was that PB provides a wider range of appraisal options than simply asking ‘why?’, thus delivering potentially greater flexibility and potency when reframing distressing material.

These integrated SD-PB techniques were then trained over two-face to-face sessions complemented by two weeks of self-guided home-based practice. The main focus of the SD-PB training was deliberately not on highly distressing major life events in the individual's life (although these did feature) but rather on everyday sources of stress and upset – so called, ‘daily hassles’ (Kanner, Coyne, Schaefer, & Lazarus, 1981). This is in line with cognitive theories of depression which propose that it is the propensity to process and interpret these types of everyday events in negatively dysfunctional and potentially catastrophic ways that confers much of the cognitive vulnerability to relapse (Lau, Segal, & Williams, 2004).

Clearly a key part of the SD-PB training protocol is the processing of negative emotional events. For this reason it was imperative that any comparison training condition included similar exposure to such material and was also equally plausible to participants (cf. Kross et al., 2012; Schartau et al., 2009). We therefore developed an Overcoming Avoidance (OA) comparison protocol that involved comparable processing of emotional material, though without the SD-PB instructions, and framed within the rationale that overcoming your urge to avoid thinking about difficult experiences has potential therapeutic benefits (Wells, 2013).

Participants comprised individuals with a diagnosis of major depressive disorder currently in remission. We only included those with a recurrent course comprising at least three previous major depressive episodes as recurrent depression is most closely associated with heightened sensitivity to, and dysfunctional appraisals of, everyday negative events (Teasdale, 1988). This also matches the inclusion criteria for trials of intensive preventive clinical interventions such as MBCT (Kuyken et al., 2016).

In terms of outcomes, we examined both the cognitive and affective effects of SD-PB training versus OA training, using standardized self-report measures of self-distancing and perspective broadening alongside targeted rating scales based on earlier work (Kross et al., 2012). We also looked at changes in residual symptoms of depression as a marker of depressive risk. Obviously, the gold standard depression outcome for a sample currently in remission would be to evaluate the impact of training on the likelihood of depressive relapse over time. However, this is inappropriate for the early stage evaluation of a clinical technique (which is necessary prior to progression to a clinical trial; Medical Research Council, 2000) and residual symptomatology is widely accepted as a useful surrogate measure of relapse risk (Beshai, Dobson, Bockting, & Quigley, 2011; Judd et al., 1998).

We had hypotheses pertaining to two sets of effects of training as follows:

Within-training effects

**Hypothesis 1**. That those trained in SD-PB strategies, relative to OA, would report reduced distress when those strategies were explicitly applied during training in response to everyday, personal negative memories, to novel negative emotional events recorded using a diary, and to memories of negative life-events (we included these more potent negative memories to examine the breadth of impact of the SD-PB techniques).

Outcome effects

**Hypothesis 2**. That, following training, SD-PB training, relative to OA training, would lead to improvements in self-reported self-distancing and perspective broadening on standardized questionnaires.

**Hypothesis 3**. That, following training, those who had received SD-PB training, relative to OA training, would report reduced self-reported distress to negative emotional events recorded in a diary (Hypothesis 3a) and a greater reduction in distress relative to baseline to negative life event memories (Hypothesis 3b), this time in the absence of explicit instructions to apply the training strategies.

**Hypothesis 4**. That SD-PB training, relative to OA training, would lead to a reduction in residual symptoms of depression relative to baseline.

**Hypothesis 5**. That, following training, those who had received SD-PB training, relative to OA training, endorse more functional and positive cognitive evaluations of everyday negative memories.

## 1 Method

### 1.1 Participants

Based on a mixed within-between groups interaction medium effect size of *f* = 0.25 derived from a between groups medium effect between remitted depressed and never-depressed participants of *d* = 0.5 (Fresco et al., 2007; Hill, 2014), a power calculation with α = 0.05 with 80% power indicated a required sample size of *n* = 13 per group for the intervention to approximately normalize performance on these measures in a remitted sample.

We therefore recruited twenty-six participants (mean [*SD*] age = 50.81 [12.10] years; 19 females) with recurrent (≥3 previous episodes) major depressive disorder (MDD), currently in remission, via advertisements in local newspapers and health centers asking for volunteers to help with psychological research. MDD diagnosis and history (including absence of a current major depressive episode), and other Axis 1 psychiatric comorbidity, according to the Diagnostic and Statistical Manual for Mental Disorders (4th edition-text revision; *DSM-IV-TR*: American Psychiatric Association, 2000), were determined using the Structured Clinical Interview for the *DSM-IV* Axis I Disorders Clinician Version (SCID, Version 2.0-revised; First, Gibbon, Spitzer & Williams, 2002). Exclusion criteria were a current diagnosis of substance dependence or abuse, a history of psychosis or manic episodes, and organic brain injury. No participants were excluded on these bases. The SCID was administered in a separate assessment session within 6 weeks prior to the first study session. Depression remission status was then confirmed in each study session. Following SCID assessment, participants were randomly allocated to either the Self-Distancing and Perspective Broadening (SD-PB; *n* = 13) or Overcoming Avoidance (OA; *n* = 13) training conditions using a computerized minimization procedure overseen by an independent statistician stratified by score (above or below the cut-off score demarcating the depressed (≥10) and non-depressed (<10) ranges; Shaw, Vallis, & McCabe, 1985) on the Beck Depression Inventory (BDI; Beck, Rush, Shaw, & Emery, 1979).^2^

### 1.2 Procedure

This study was approved by the University of Cambridge Research Ethics Committee and was carried out in accordance with the provisions of the World Medical Association Declaration of Helsinki. Participants were informed that the study was evaluating two different approaches to responding to emotional memories, and that they would be randomly allocated to complete training in one of these approaches. Participants underwent a pre-training baseline assessment and were subsequently randomly allocated to receive two weeks of training on either SD-PB or OA followed by a post-training outcome evaluation comprising a post-training assessment and completion of a 1-week diary measure. Participants provided written informed consent and were paid an honorarium of £6 per hour for their time.

### 1.3 Pre-training baseline assessment

We acquired baseline data on a number of standardized self-report measures both to characterize the sample and for use in evaluating the outcome of the training. Our symptom measures included the BDI (also administered at the start of training Session 2 in order to track depressed mood) and the Spielberger State Trait Anxiety Inventory (STAI; Spielberger, Gorsuch, Lushene, Vagg, & Jacobs, 1983), a widely used and psychometrically robust measure of trait (how the person generally feels) and state (how the person feels right now) components of anxious mood.

We also wanted to include standardized measures of self-distancing and perspective broadening. At the time of study design, the best candidate for perspective broadening was the 4-item Perspective Broadening subscale of the Cognitive Emotion Regulation Questionnaire (CERQ-PB; Garnefski, Kraaij, & Spinhoven, 2002). The CERQ-PB items probe the ability to contextualize negative events within a wider frame of reference. The items are rated on a 5-point Likert scale from 1 (*almost never*) to 5 (*almost always*). The CERQ-PB has good internal reliability, Cronbach's α = 0.82 (Garnefski et al., 2002). The best candidate for self-distancing was the 11-item Decentering subscale of the Experiences Questionnaire (EQ-DC; Fresco et al., 2007). The EQ-DC evaluates the self-reported ability to disengage from troublesome mental content and take a more accepting stance towards it. The EQ has good internal consistency, Cronbach's α = 0.81, and construct validity (Fresco et al., 2007). Both the EQ-DC and CERQ-PB has been used previously with remitted-depressed participants, with findings indicating a relatively impaired ability to decenter compared to never-depressed controls, with medium to large effect sizes (Fresco et al., 2007; Hill, 2014).

During the Baseline session, participants were also asked to generate five autobiographical memories of important negative life events. As already noted, we wanted to utilize negative life-event memories as well as everyday memories to provide a more challenging training context for the SD-PB techniques. We also included such memories in our outcome assessment to examine whether the benefits of SD-PB training extended to more difficult personal material. Participants were asked to generate memories of life events that had caused distress at the time and continued to cause distress upon recollection. Examples included the death of loved one, the breakup of a significant relationship, serious accidents and illnesses, assaults, and abuse. Each memory was rated on Likert scales from 1 = *not at all distressing* to 7 = *extremely distressing*, for both distress at the time of the original event and current distress when thinking about the event. The two memories with the most comparable levels of distress were selected for use in evaluating the outcome of the training and the remaining three were set aside for use as training material. For each of the two memories selected for outcome evaluation, participants completed the Impact of Event Scale (IES; Horowitz, Wilner, & Alvarez, 1979) in relation to each event. The IES is a 15 item self-report measure of psychological distress associated with identified events. It contains 2 subscales: Intrusion which refers to intrusive thoughts, feelings, imagery or nightmares about the event; and Avoidance which refers to avoidance of feelings, situations, ideas associated with the event. The items are rated on a six point scale detailing the extent to which they have been true over the previous week from 0 (*not at all*) to 5 (*often*). The IES has good internal consistency, Cronbach's as ranging from 0.79 to. 92, and test-retest reliability, ranging from 0.79 to 0.89 (Corcoran & Fischer, 2013). The IES was employed at baseline and post-training to evaluate changes in distress as a function of training.

### 1.4 Self-distancing and perspective broadening (SD-PB) training

SD-PB training took place over two weeks, with two face-to-face sessions (one each week) and daily home-based training in the form of scenario-based memories and diary tasks. Twelve of the 13 participants completed both training sessions, and we achieved 85% participant adherence to the homework exercises. The first training session began by introducing participants to the SD-PB techniques using an instructional video narrated by one of the authors (TD). The video introduced the ideas of loss of perspective in depression and presented the rationale for training in self-distancing and in expanding perspective to consider ‘the bigger picture’. The experimenter (EH) then asked each participant to think of a recent upsetting event from their everyday life (e.g., an argument with a friend, partner or colleague, making a mistake at work). She then guided the participant through the basic SD-PB techniques in relation to this event using a standardized semi-structured script in order to familiarize the participant with the core principles of the training.

This guided exercise initially detailed the SD technique (cf., Kross et al., 2012): participants were asked to recall all the details of the selected event and ‘build a mental picture of it playing out again, seeing the events unfold’. When ready, they were asked to imagine that the memory they had in their mind was taking place on a theatre stage and that they were playing themselves as one of the actors. Once they had a detailed and vivid image in mind, they were then asked to imagine walking off of the stage and up into a balcony box, and then to view the memory again from the new vantage point, looking down on themselves on the stage. Once participants felt confident in imagining the event and with the method of SD using the imagined balcony box, they were introduced to the next step.

**Fig. 1:**
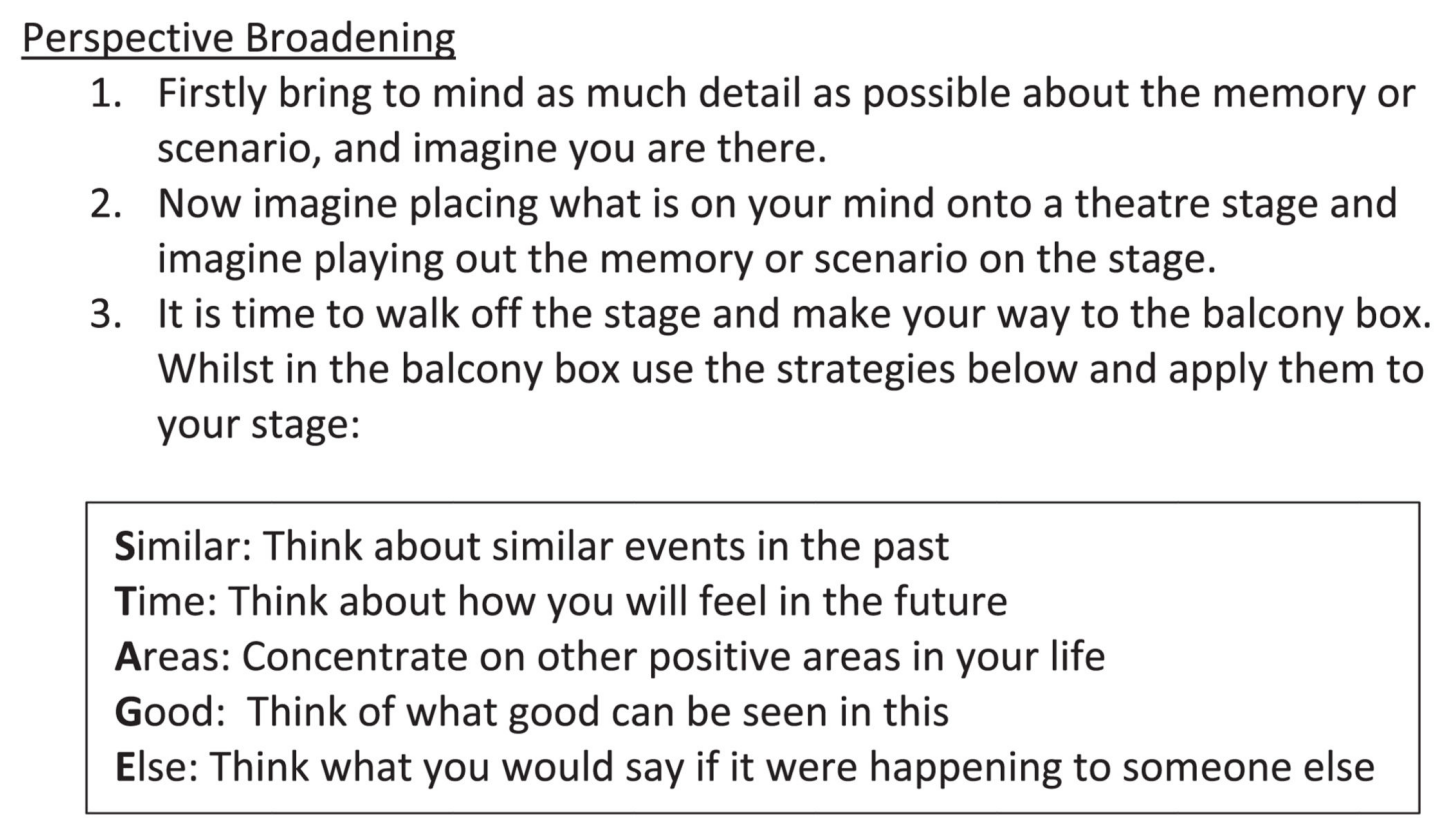
Cue card given to participants in the Self-Distancing and Perspective Broadening condition.

This second step introduced five PB strategies. Each strategy required participants to broaden their evaluation of the event along a different perspective dimension. As a mnemonic aide, the strategies were labelled such that their initial letters made up the acronym ‘STAGE’ (summarized on a cue card given to each participant; see Fig. 1). The five strategies were: ‘*Similar*’ which asked participants whether they could think of similar events in their past to the event in question but that were less distressing, or even positive (e.g., if the event was an argument with a partner, are there more positive experiences with that person that can be brought to mind); ‘*Time*’ which prompted participants to think about how they will feel about the event at different points in the future once more time has elapsed; ‘*Areas*’ which asks participants to reflect on their life as a whole and acknowledge the more positive areas that may offer a contrast with the event in question; ‘*Good*’ which asks participants to consider whether there were any aspects of the event itself that were relatively less negative or maybe even would turn out to have some more positive consequences (e.g., for the afore-mentioned argument, did something constructive nevertheless come out of it, even if it was only awareness of another's point-of-view); and ‘*Else*’ which prompts the participant to think about either what they would say to a close friend who was going through the same thing if they wanted to help that friend to gain perspective on the event, or what such a friend might say to them.

During this exercise participants were assisted with applying each strategy to their pre-selected event. They were also encouraged to elaborate on each strategy as best they could with a visualization exercise in which they re-scripted the depiction of the event on the theatre stage from their self-distanced vantage point in line with the strategy they were applying. For example, the suggested elaboration for the ‘*Similar*’ strategy was to switch the distressing event for a similar less negative or positive memory playing out on the stage.

Once participants felt comfortable with the SD technique and with the five PB strategies, they commenced training with these techniques using memories of everyday negative events that they had found upsetting. The recollection of these everyday events was prompted using a series of written scenarios based on those used by Teasdale et al. (2002). In line with Teasdale et al.'s (2002) method, participants were asked to try to think of a memory similar to the situation portrayed in the scenario and to then apply the SD-PB techniques to that memory. The scenarios were chosen to portray events that people susceptible to depression are likely to be particularly sensitive to, resulting in the activation of depressogenic themes such as failure, lack of self-worth and so forth (Beck et al., 1979). Accordingly, scenarios covered events such as someone not acknowledging you in the street, burning dinner, or feeling left out at a party. The full set of scenarios is available on request.

For each of five scenarios participants first sought to generate a similar memory, if they were unable to do this they were told to work with the scenario itself (cf. Teasdale et al., 2002). Participants then visualized the memory on the stage and self-distanced from it by imagining ascending to the balcony box. They then worked through the five PB strategies. For each scenario participants rated whether they noticed a change in their distress after applying the SD-PB techniques (on a 20-point Likert scale from –10 = ‘*decreased distress*’ to +10 = ‘*increased distress*’; cf., Kross et al., 2012). Having spent 50 min on this in-session everyday negative memory training, participants were asked to continue the training at home, using their cue card, with one new scenario-cued memory each day (seven in total, provided in a booklet along with the rating scales) for a week until the second face-to-face session. This took approximately five minutes each day. This first session (and the two subsequent sessions) ended with a positive memory recall exercise to enhance mood.

Session 2, one week later, began with a review of the home-based training followed by 45 min of training with a further five everyday negative memories, again cued by scenarios. Participants were then asked to apply their SD-PB skills to the three negative life event memories that they had generated at pre-training and that had been selected for use during the training, and complete the same change in distress rating as for the everyday memory training.

Participants were then provided with instructions for further home-based training between Session 2 and the outcome assessment, this time focusing on applying the SD-PB techniques to *newly encountered* everyday upsetting events. They were asked to complete an everyday emotional events diary twice a day, recording anything significantly upsetting that had happened. For each identified event in the diary, participants were asked to use their cue card to explicitly work through the SD and PB strategies. Prior to completing the diary, participants retrospectively rated their distress at the time that the event occurred earlier that day and, after diary completion they rated their current distress about the event using Likert scales from 1 *not distressing* to 9 *very distressing* (cf. Kross et al., 2012).

### 1.5 Overcoming avoidance (OA) training

The OA training procedure emphasized overcoming avoidance pertaining to distressing memories and events. Participants were educated on the role of avoidance in maintaining psychological disturbance, and on how reducing avoidance of negative material (by actively retrieving negative memories and letting yourself experience the flow of emotion that is naturally aroused by the memory) can yield benefits for emotional health. OA training was kept as close as possible to the SD-PB training structure and utilized the same stimuli. As in the SD-PB condition, OA participants engaged in their memories for 50 min in Session 1 and 45 min in Session 2, and completed five minutes of home-based exercise each day for a week. The key difference between the two training conditions was that individuals in the OA group were not asked to self-distance or broaden their perspective for each memory but rather to “build a mental picture of it playing out again, seeing the events unfold”. To that end, in the first session the OA group were shown an alternative instructional video that highlighted the benefits of overcoming avoidance about distressing situations and were not instructed to apply SD-PB techniques to the negative life event memories, the everyday negative memories cued by the scenarios, nor the everyday negative events recorded in their diary. Twelve of the 13 participants completed both training sessions, and we achieved 92% participant adherence to the homework exercises.

### 1.6 Post-training outcome evaluation

The final face-to-face session focused on evaluating the outcome of the training and was the same for the SD-PB training and OA training groups. All participants repeated the questionnaires from the pre-training baseline session: the BDI, STAI, CERQ-PB, and the EQ-DC. Following this, the two negative life event memories that had been rated at baseline were re-rated in terms of current distress when thinking about them and using the IES.

To evaluate the impact of SD-PB and OA on how emotional experiences were being processed, participants were presented with a last set of four scenarios to use as prompts for negative everyday memories as before. In each case, participants were asked to spend time thinking about the events at hand but again, unlike the training sessions, they were not now provided with specific instructions as to how to process the material. After reflecting on this set of everyday negative memories, participants generated five ratings indexing different aspects of how they now thought about such events following their training: ‘*the extent to which they thought about the positive aspects of the events*’; ‘*how easy it was to think of the positive aspects of the events*’; ‘*the extent to which they thought about the negative aspects*’; ‘*how easy it was to think of the negative aspects*’; and finally ‘*the extent to which they thought about the situation differently*’. Each rating was made on a 7-point ‘extent’ Likert scales from 1 = ‘*Not at all*’ to 7 = ‘*Extremely so*’. This use of bespoke measures to specifically probe thinking strategies is in line with other research in the SD and PB literatures (e.g. Kross et al., 20).

Finally, participants were then asked to complete the everyday emotional events diary and its associated ratings for a further week following this assessment session but this time, unlike during the training, there were now no specific instructions regarding how they processed the events. The participants posted the diaries back to the experimenter at the end of the week.

## 2 Results

### 2.1 Description of the sample

Two participants (one per condition) dropped out of the study after the first session. Full data are therefore reported for the remaining 24 participants, 12 per condition. All participants engaged in the assigned homework tasks, except for one participant in the SD-PB group who did not return the final outcome diary. There was no significant correlation between the number of events recorded during home-based training and any of the outcome measures for the SD-PB, *rs* (n = 10) < 0.36, *ps* > 0.30, or OA condition, *rs* (n = 11) < 0.38, *ps* > 0.22. This was also true for the number of events recorded in the everyday emotional events diary, SD-PB *rs* (n = 10) < 0.38, *ps* > 0.28, OA *rs* (n = 11) < 0.43, *ps* > 0.16. Descriptive statistics and pre-training questionnaire outcome measure data are presented in Table 1. As can be seen from the table, the groups did not differ significantly on any of these variables at pre-training. As expected, both groups showed some degree of residual depressive symptoms with the mean baseline BDI scores falling just within the “Mildly Depressed” range of >10 (Shaw et al., 1985).

In addition to MDD, we assessed other Axis 1 diagnoses on the SCID at study entry. In the SD-PB condition, 2 participants met criteria for panic disorder (PD), 3 for posttraumatic stress disorder (PTSD), 1 for specific phobia (SP), 3 for generalized anxiety disorder (GAD), and 1 for anxiety disorder not otherwise specified. In the OA condition 1 met criteria for PD, 1 for OCD, 2 for SP, 2 for GAD, and 1 for social phobia. We did not reassess diagnostic status after study entry except for depressive relapse which was assessed at each study session using the SCID. No participants relapsed into a current Major Depressive Episode across the duration of the study.

During the SCID, 12 participants (5 in OA and 7 in SD-PB) reported having completed psychological therapy in the past. Only three were able to recall which type, which was CBT for 2 participants (1 in each group) and cognitive analytic therapy for one participant in the SD-PB group. The majority of participants had received anti-depressants at some point in their life, with only one participant in the SD-PB condition and two in the OA condition reporting that they had never taken medication for mental health issues. Following random allocation, the use of concurrent treatment was evenly distributed between conditions. Antide-pressant medication was used for the duration of the study by four participants in the SD-PB condition and five participants in the OA condition. No participants were currently receiving psychological intervention.

### 2.2 Hypothesis 1: impact of SD-PB during the training

As outlined above, as an integral part of the training, participants in both conditions processed three negative life event memories (e.g., deaths or illnesses of loved ones, relationship breakups, accidents, serious arguments) in Session 2 and a series of everyday negative memories (cued by scenarios) used as training material across Session 1, Session 2 and the home-based training between Sessions 1 and 2. They also completed a week-long diary, recording new everyday negative events following Session 2. Performance across each of the three negative life event memories was comparable and the data were therefore averaged for each participant. This was also the case for the scenario-cued everyday negative memories used in training and again the data were averaged. These mean life event and mean everyday negative memory ratings are presented in Table 2, along with the ratings of distress recorded in the home-based training.

As can be seen from Table 2, in support of our first hypothesis, the SD-PB group reported significantly greater reductions in distress when explicitly applying their trained strategies than did the OA training group for the negative life event memories, *t*(22) = 6.27, *p* < 0.01, Hedges' *g* = 2.56, and the everyday negative memories, *t*(22) = 5.58, *p* < 0.01, Hedges' *g* = 2.31.

For the everyday negative events recorded in the home-based training diary between Sessions 1 and 2, the SD-PB group reported an average of 3.55 (SD = 1.63) events and the OA group 4.83 (SD = 3.22) events. There was no significant difference between the groups on the number of events reported, *t*(16.63) = 1.23, *p* = 0.24, Hedges' *g* = 0.50. Events included worries, problems at work, and minor accidents. We compared self-report ratings of current distress following thinking about the event in line with the training instructions while recording it in the diary, covarying ratings of retrospectively-rated distress at the time that the event occurred to ensure that any differences were not simply a function of differences in the distress originally elicited by the events (see Table 2 for both ratings). As predicted, there was a significant group difference, *F*(1, 21) = 5.81; *p* < 0.05, $\eta_{p}^{2}=0.24,$ with the SD-PB group reporting relatively less distress.

### 2.3 Outcome of training

#### 2.3.1 Hypothesis 2: standardized self-report measures of SD-PB

Table 1 presents the pre- and post-training scores for the CERQ-PB and the EQ-DC outcome measures. Repeated measures ANOVAs revealed the predicted significant interaction of Time (pre-vs. post-training) and Group (SD-PB vs. OA) for both CERQ-PB, *F*(1,21) = 5.29, *p* = 0.03, $\eta_{\text{p}}^{2}=0.20,$ and EQ-DC, *F*(1,21) = 15.85, *p* < 0.01, hp = 0.44. Paired t-tests for each group separately were conducted to clarify the nature of the changes within each group. These revealed no significant changes over time for the OA group, *t*s<0.71, *p*s>0.49, while the SD-PB group showed significant improvement on both the CERQ-PB, *t*(11) = 3.52, *p* < 0.01, Hedges' *g* = 0.48, and EQ-DC measures, *t*(11) = 6.47, *p* < 0.01, Hedges' *g* = 1.14.

#### 2.3.2 Hypothesis 3: everyday negative events and negative life event memories

For the everyday events diary completed as an outcome measure (during the week following Session 2), the SD-PB group reported an average of 3.45 (SD = 2.07) events and the OA group 4.83 (SD = 3.27) events. The types of event were similar to those reported during training. There was no significant difference between the groups on the number of events reported, *t*(20) = 1.20, *p* = 0.25. As for the within-training diary data, we compared self-report ratings of current distress following thinking about the event while recording it in the diary, covarying ratings of retrospectively rated distress at the time that the event occurred (see Table 3 for both ratings). There was the predicted significant group difference, *F*(1, 19) = 4.24; *p* < 0.05, $\eta_{p}^{2}=0.18,$ with the SD-PB group reporting relatively less distress.

For the mean ratings across the two negative life event memories rated at pre- and post-training, a mixed model ANCOVA on ratings of current distress experienced to the memories, covarying the distress ratings for the time that the event occurred (see Table 3 for data), revealed no significant main effects of time or group, *F*s < 1, and a medium effect for the time x group interaction, *F*(1, 20) = 3.10; *p* = 0.09, $\eta_{p}^{2}=0.13,$ with the SD-PB group tending to show a greater reduction in distress relative to baseline compared to the OA group, though this trend was non significant. Similar repeated measures ANOVAs were conducted on the Impact of Event Scale subscales pre- and post-training (Table 2). The Intrusion subscale scores revealed a main effect of Time, *F*(1,22) = 7.56, *p* = 0.01, $\eta_{p}^{2}=0.26,$ with levels of intrusions decreasing from baseline to post-training, no effect of group, F < 1, nor a group x time interaction, *F*(1,22) = 2.82, *p* = 0.11, $\eta_{p}^{2}=0.11.$ The Avoidance subscale scores revealed no main or interactive effects, *F*s < *2.59*, *p*s > .*12*.

#### 2.3.3 Hypothesis 4: residual symptoms of depression

Table 1 presents the baseline, Session 2, and post-training BDI scores, which were used to index residual depressive symptoms. A mixed model ANOVA comparing BDI scores at the three time points for the two groups revealed a significant interaction of Time by Group, *F*(2,40) = 3.70, *p* = 0.03, $\eta_{p}^{2}=0.16,$ in line with our hypothesis. Follow-up ANOVAs indicated that change in residual depressive symptoms from baseline to Session 2 differed significantly between SD-PB and OA conditions, *F*(1,20) = 5.50, *p* = 0.03, $\eta_{p}^{2}=0.22,$ as did change from baseline to post-training, *F*(1,22) = 5.93, *p* = 0.02, $\eta_{p}^{2}=0.21.$^3^ There was no significant dif ference between the groups in change from Session 2 to post-training, *F*(1,20) = 0.04, *p* = 0.84, $\eta_{p}^{2}=0.002.$ Follow-up within-subjects tests were conducted to provide clarity around the nature of changes for each group. They showed that the SD-PB group evidenced a significant reduction in residual symptoms of depression between baseline and Session 2, *t*(10) = 2.38, *p* = 0.04, Hedges' *g* = 0.20, with scores then stabilizing such that there was no significant change between Session 2 and post-training, *t* < 1. There were no significant changes over any of the time points for the OA group, *t*s<1.40, *p*s>0.20.

#### 2.3.4 Hypothesis 5: thinking strategies to scenario-cued everyday memories

A MANOVA for the mean scores of the five bespoke ratings applied to how participants thought about the everyday scenariocued memories (see Table 3) at post-training revealed a significant multivariate difference between the two groups, Wilks' Lambda = 0.48, *F*(1, 22) = 3.87, *p* = 0.02, $\eta_{p}^{2}=0.52.$ Analyses on the univariate output were Bonferroni corrected for multiple testing (α = 0.05/5 = 0.01). The findings showed that the SD-PB group scored significantly higher than the OA group for ‘*the extent to* which they thought about the positive aspects of the situation ’, and the ‘*extent to which they thought about the situation differently*’, *t*s > 3.13, *p*s < 0.005. There was a large effect for ‘*how easy it was to think of the positive aspects of the situation*’, but this became non significant after Bonferroni correction, *t*(22) = 2.26, *p* = 0.03, Hedges' *g* = 0.92. There were no significant univariate group differences for ‘*how easy it was to think of the negative aspects*’, nor ‘*to what extent they thought about the negative aspects*’, *ts* < *1.43*, *ps* > *0.16*.

## 3 Discussion

The current study investigated the impact of a novel cognitive training methodology designed to foster the ability to decenter or self-distance from distressing material and to adopt a broader psychological perspective when evaluating that material: Self-Distancing and Perspective-Broadening (SD-PB) training. We tested five hypotheses relating to the impact of applying SD-PB both during training and as an outcome of training.

Our first hypothesis was that during training when participants are being instructed to apply their allocated strategies (SD-PB or OA), SD-PB would be superior to OA in its ability to reduce distress during the processing of emotive personal material. This was supported with consistently large effect sizes (Cohen, 1988) for pre-selected significant life-event memories, memories of more minor everyday negative events (cued by scenarios), and novel everyday events recorded in a diary – daily hassles (Kanner et al., 1981). This confirms the findings from earlier work (Kross & Ayduk, 2011) that self-distancing from distressing information can be beneficial (cf. Kuyken & Moulds, 2009) if participants are provided with functional ways to process the information from a self-distanced stance, in this case using appraisals to broaden perspective.

The remaining four hypotheses examined the outcomes of SD-PB (versus OA) training, with the aim of evaluating intrinsic shifts in processing style and impact on depressive risk. In support of Hypothesis 2, those receiving SD-PB training showed significant improvements on standardized self-report measures of perspective broadening and the self-distancing aspect of decentering, while there was no support for such changes in the OA group, and the magnitude of the difference in these effects between the groups was significant. In support of Hypothesis 3a, those receiving SD-PB training reported reduced negative mood and improved positive mood when processing novel daily hassle events recorded using a diary procedure. These findings mirror the within-training diary results described above (in support of Hypothesis 1), but this time in the absence of explicit instructions to process the material using a particular strategy. We failed to support Hypothesis 3b, finding no significant evidence that those trained in SD-PB experienced relatively greater reductions in distress when processing negative life-event memories relative to those trained in OA (although there was a trend for a medium effect in the anticipated direction), compared to baseline. This contrasts to the within-training findings (Hypothesis 1) for life event memories. Similar findings emerged for the Impact of Event Scale ratings to the life event memories, where the only significant effect was an overall reduction in levels of memory intrusions, as a function of training, across all participants. We also found no effect of time on avoidance, which was surprising given the key aim of the OA condition. This lack of effect may reflect that more extensive, repeated exposure over a longer duration of time using monitoring of distress (as in exposure therapy; Foa, Hembree, & Rothbaum, 2007) may be needed to reduce entrenched avoidance habits, which was beyond the scope of this low-intensity training protocol.

These post-training life event memory data provide no support for SD-PB training being differentially helpful, relative to training in OA, in changing the processing of memories of major life events (e.g. death of a loved one) when participants are no longer being explicitly instructed to apply the SD-PB strategies. In many ways this is unsurprising as the SD-PB strategies are targeted at diluting the effects of everyday negative experiences and daily hassles, where shifts in perspective are anticipated to have a marked and immediate impact with the aim of reducing the likelihood that such events will precipitate downward spirals of negative thinking and feeling (Kanner et al., 1981). It is important to note, nevertheless, that processing of life event memories did still improve following SD-PB training, in terms of reduced intrusions of such memories on the IES. However, this was also the case for participants trained in OA and could either reflect the fact that both training protocols are beneficial in reducing intrusions, some non-specific effect of exposure to a memory protocol, and/or retesting on the same memories.

The data provided support for Hypothesis 4 with SD-PB training, relative to OA training, leading to a decrease in residual symptoms of depressed mood compared to baseline, measured with the BDI, with mean scores reducing from just inside the “mildly depressed” range to just inside the “non-depressed” range in the SD-PB group (Shaw et al., 1985) and by an average of three points on the BDI. Residual symptoms in those with recurrent depression and a history of multiple previous episodes are a significant predictor of later relapse and thus a useful surrogate marker of relapse risk (Beshai et al., 2011; Judd et al., 1998). The observed decrease in residual depressive symptoms occurred in the week between Session 1 and Session 2, and was maintained at the post-training evaluation one week later. The plateau in effect on residual depressive symptoms between Session 2 and post-training may reflect floor/ceiling effects on depressive symptoms in an already remitted sample, or the fact that larger effects are generated when the participant is explicitly instructed during practice of the techniques, as occurred between Sessions 1 and 2. Participants were also required to work with more personally poignant memories after Session 2, which is likely to have been harder and may therefore have reduced the effectiveness of the skills. A larger trial with longer follow-up and sample with clinical levels of depressive symptoms will now be needed to examine any durable and clinically significant effect of the protocol on depression symptoms. Nevertheless, the reduction in scores on the BDI, although small, was in line with the ≥3 point change ‘rule of thumb’ from the National Institute for Health and Care Excellence (NICE) to characterize a minimal clinically meaningful change, potentially indicating some change in depressive risk (National Collaborating Centre for Mental Health [NCCMH], 2004). These findings provide a promising platform for further evaluation of the SD-PB protocol for depression.

Our final hypothesis (Hypothesis 5) explored whether SD-PB training, relative to OA training, was associated with differences in the post-intervention thinking strategies that participants reported using when processing negative everyday memories. We found that, relative to OA, those trained in SD-PB reported significantly differentially enhanced positive reappraisal of the memories and the ability to 'think about them differently'. We found no support for SD-PB differentially altering the processing of negative components of the memories. This pattern is perhaps unsurprising given the focus of SD-PB on identifying and applying positive reappraisals that broaden perspective, as opposed to challenging and reappraising negative material *per se*. These findings using bespoke measures of processing change complement the similar findings on the standardized self-report measures presented above and suggest that SD-PB does bring about a significant shift in the way that at least some distressing experiences are negotiated.

Taken together the present data provide preliminary evidence that systematic training in self-distancing and perspective broadening can provide currently-remitted patients with recurrent depression with important skills to reduce reactive distress and enhance functional cognitive processing of both remembered and newly encountered everyday negative experiences. Allied with the small but significant beneficial impact of such training on residual symptoms of depression relative to OA, this suggests that SD-PB has promise as a stand-alone or adjunctive training regime for use in clinical practice to promote resilience and potentially to reduce relapse risk in those with a history of depression. Cognitive training programmes commonly seek to influence explicit or implicit biases in cognitive processes. While implicit training programmes such as cognitive bias modification (CBM; MacLeod & Mathews, 2012) have been helpful in shifting low-level bias (e.g., in attention to threatening information), more durable cognitive processes and skills such as perspective broadening are thought to require more explicit training (Dalgleish & Werner-Seidler, 2014). In this regard, SD-PB can be considered alongside other protocols such as autobiographical memory specificity training (Raes, Williams, & Hermans, 2009) as part of a broad family of low-intensity cognitive interventions which use repeated practice of new cognitive skills to mitigate cognitive deficits in those who suffer mood difficulties (see Hitchcock, Werner-Seidler, Blackwell, & Dalgleish, 2017). However, the SD-PB protocol does arguably improve upon current low-intensity cognitive interventions by explicitly targeting multiple cognitive processes thought to promote depressive relapse.

The current study design sought to isolate self-distancing and perspective broadening techniques which form one aspect of larger treatment protocols, particularly MBCT. A change in perspective on the self is proposed to be an active therapeutic component of MBCT (for discussion see Hölzel et al., 2011), and our findings indicate that self-distancing and perspective broadening skills more specifically may form key mechanisms through which MBCT has therapeutic effect. Further exploration of self-distancing and perspective broadening skills as mediators of MBCT therefore seems warranted, in addition to further assessment of the SD-PB protocol as a standalone, low-intensity intervention which is less cognitively demanding than MBCT and can be delivered by low-intensity trained therapists.

A particular strength of the study is the inclusion of an active control condition (cf. Kross et al., 2012) – Overcoming Avoidance Training – that ensured that control participants were exposed to, and processed, comparable amounts of emotive material to the SD-PB group. Assistance in overcoming avoidance is itself a core component of cognitive-behavioral interventions for emotional disorders and so the inclusion of OA as a control here sets an appropriately high bar against which to evaluate the impact of SD-PB training.

However, the current study also raises a number of methodological issues that merit discussion. Firstly, the sample size was modest, although it was in line with pre-study power calculations and consistent with advice surrounding platform studies of novel clinical interventions (MRC, 2000). Despite the modest sample size, almost of all of the hypothesized effects of SD-PB were supported and, where there was no support (e.g., for the predicted differential improvements in IES scores) the effects were sufficiently small to suggest that insufficient statistical power was not an issue. There was only one instance where a larger sample may have allowed us to detect potentially important effects in the data at the traditional level of significance. This was the change in distress to negative life-event memories from baseline to post training where we found a medium but non significant effect for an interaction in the expected direction. The fact that the sample size was insufficient to provide a proper evaluation of this issue must therefore be regarded as a study limitation.

The second issue concerns the decision not to include a healthy comparison group. There were two reasons behind this choice. Firstly, SD-PB is aimed at enhancing cognitive processing of everyday negative information in individuals with recurrent depression who are at risk of future episodes. This is because we know that dysfunctional processing of such information is one of the major precipitants of the downward spirals of thinking and feeling that initiate such relapses (Lau et al., 2004). There is no comparable theoretical rationale for SD-PB training being of benefit for healthy participants. Secondly, cross-sectional studies of SD (Kross et al., 2012) and PB (Schartau, 2006) suggest that these techniques indeed accrue little added benefit for healthy individuals. For example, in their healthy control group, Kross et al. (2012) found no significant advantage of SD over immersion when processing a distressing memory, and a small between-condition effect size, Cohen's *d* = 0.20 (p. 564).

A third issue is the reliance on self-report measures. Although we followed Kross et al. (2012) in using both standardized questionnaires and bespoke Likert scale ratings, the outcomes would have been strengthened if we had also followed Kross et al.'s lead and included an objective, experimental measure of SD and PB (e.g., the self-distancing task developed by Shepherd, Coifman, Matt, & Fresco, 2016). That said, the use of a plausible active control – Overcoming Avoidance training – that was presented to participants with a comparably compelling rationale as SD-PB training and that itself led to benefits in the way material was processed post-training (e.g. reduced life event memory intrusions) means that response bias – a common criticism levelled at self-report measures – is less likely to account for the current results. Finally, the present study assessed the impact of SD-PB training only up to a week after training had finished (the diary component). Clearly, it will now be important to evaluate the longer-term impact of this kind of training in a randomised controlled trial to ensure that the effects are durable. A future trial should aim to improve on single item measures of SD-PB strategies, examine the impact of the frequency of strategy use on outcomes to inform the further development of the protocol, and begin to separate the individual effects of reappraisal and self-distancing elements of the protocol. This situation mirrors the early studies on CBM (MacLeod, Koster, & Fox, 2009), with later work extending the investigations of impact over longer-durations (MacLeod & Mathews, 2012).

In summary, the current study shows that systematic training in SD-PB has beneficial effects on the cognitive and affective processing of negative autobiographical material and can bring about small but significant reductions in residual symptoms of depression in individuals with recurrent depression who are not currently in episode. This testifies to SD-PB's potential as a low-intensity stand-alone or adjunctive intervention for future clinical application.

## Figures and Tables

**Table 1:** Mean (SD) descriptive statistics and pre-training outcome questionnaire data for the SD-PB and OA training groups.

Measure	SD-PB (*n* = 12)	OA (*n* = 12)	Baseline test and significance statistics
Age	50.08 (13.87)	51.75 (11.73)	*t* < 1, *p* = 0.75, Hedges' *g* = 0.13
Gender (Male:Female)	2:10	4:8	*Fisher's Exact*, *p* = 0.64
Median no. previous MDEs	TMTC/ID	5	
BDI Baseline	11.36 (9.60)	10.91 (7.18)	*t* < 1, *p* = 0.80, Hedges' *g* = 0.05
Range	1–30	3–27	
BDI Session 2	9.36 (8.90)^a^	13.09 (7.66)	
Range	0–26	0–26	
BDI Post-training	8.36 (9.26)	12.45 (8.54)	
Range	1–26	3–31	
STAI-Trait Baseline	46.92 (11.36)	48.58 (9.68)	*t* < 1, *p* = 0.70, Hedges' *g* = 0.15
STAI-Trait Post-training	44.64 (12.14)	48.58 (9.92)	
STAI-State Baseline	37.75 (11.78)	38.33 (9.41)	*t* < 1, *p* = 0.90, Hedges' *g* = 0.05
STAI-State Post-training	37.25 (11.25)	42.33 (12.91)	
CERQ-PB Baseline	12.17 (4.15)	12.92 (3.97)	*t* < 1, *p* = 0.66, Hedges' *g* = 0.18
CERQ-PB Post-training	14.55 (3.45)	13.00 (4.41)	
EQ-DC Baseline	39.50 (7.38)	39.00 (11.75)	*t* < 1, *p* = 0.90, Hedges' *g* = 0.05
EQ-DC Post-training	51.18 (10.27)	41.58 (10.02)	

*Note.* MDE = Major Depressive Episode; TMTC/ID = Too many too count or indistinguishable from each other; BDI=Beck Depression Inventory; STAI-State and STAI-Trait = Spielberger State Trait Anxiety Inventory-Trait measure; CERQ-PB=Cognitive Emotion Regulation Questionnaire: Perspective Broadening subscale; EQ-DC = Experiences Questionnaire-Decentering subscale.

a BDI data for one participant in the SD-PB group were missing for Session 2.

**Table 2:** Mean (SD) within-training measures for the SD-PB and OA training groups.

Measure	SD-PB	OA
Change in distress for the negative life event memories	–3.42 (2.84) ^a^	2.83 (1.96) ^a^
Change in distress for the everyday negative memories	–1.76 (1.50) ^a^	1.21 (1.03) ^a^
**Everyday emotional events recorded during home-based training**^b^		
Distress at the time rating	4.17 (0.51)	4.74 (0.63)
Distress now (after filling in diary) rating	2.67 (1.02)	4.13 (0.92)
**Mean rating of usefulness of SD-PB strategies**^c^		
Similar	4.30 (1.46)	
Time	4.79 (1.24)	
Areas	4.62 (1.43)	
Good	4.39 (1.04)	
Else	5.16 (0.85)	

*Note*.

a Differed significantly from zero

b Completed between Sessions 1 and 2.

c Rated by participants on a 7 point Likert scale from 0 = not at all useful to 7 = extremely useful. Mean calculated across ratings for use with negative life event memories and everyday negative memories. There was no significant difference in the reported usefulness of each strategy, *F*(4, 10) = 2.23, *p* = 0.08, $\eta_{p}^{2}=0.18.$.

**Table 3:** Outcome measures for the SD-PB and OA training groups.

Measure	SD-PB means	OA means
**Everyday negative memories**		
Extent of negativity	4.65 (0.97)	5.13 (0.64)
Ability to think about negative aspects	4.85 (1.19)	5.27 (0.76)
Extent of positivity	3.98 (0.97)	2.67 (0.60)
Ability to think about positive aspects	3.83 (1.13)	2.85 (0.99)
Ability to think differently	4.78 (1.03)	3.31 (1.25)
		
**Negative life event memories**		
Distress at time	6.46 (0.75)	6.40 (0.70)
Distress Session 1	4.17 (1.50)	4.16 (1.50)
Distress Session 3	3.71 (1.49)	4.55 (1.21)^a^
IES-I Baseline	12.04 (6.35)	9.04 (5.23)
IES-I Post	7.21 (7.05)	7.88 (5.87)
IES-A Baseline	12.79 (8.16)	8.21 (5.86)
IES-A Post	8.25 (8.59)	7.96 (7.55)
		
**Everyday emotional events diary completed the week after training**		
Distress at the time rating	5.17 (1.01) ^b^	5.02 (0.82)
Distress now (after filling in the diary) rating	3.17 (1.40) ^b^	3.72 (1.29)

*Note*.

a One participant in the OA group did not provide memory distress ratings.

b One participant in the SD-PB group did not return the outcome diary measure and one participant in the same group returned the diary but did not report any negative events. IES-I/A = Impact of Event Scale-Intrusion/Avoidance subscales (Horowitz et al., 1979).

## Footnotes

1 It is important to note that although MBCT does not promote active reappraisal of the content of mental events, it does involve reappraisal of the phenomenology; for example, by thinking of thoughts and feelings as mental events rather than ‘truths’, and fostering an attitude of curiosity and equanimity towards these experiences as opposed to one of reactive aversion.

2 The original version of the BDI was used here for legacy reasons to enable consistency with previous data within the research group.

3 In the SD-PB condition, two participants experienced no change in BDI from pre to post-training, three experienced an increase in BDI (two by 1 point, one by 2 points) and seven experienced a decrease in BDI (one by 2 points, three by 3 points, and three by 6 or more points). One SD-PB participant did experience a decrease of 16 points on the BDI, however, when this participant was excluded as an outlier, the hypothesised interaction remained significant, *F*(2,38) = 3.29, *p* = 0.048, $\eta_{p}^{2}=0.15.$ In the OA condition, one participant experienced no change in BDI, eight experienced an increase in BDI (one increased by 1 point, two by 2 points, and five by 4 or more points). Three experienced a decrease in BDI (one by 1 point, one by 5 points, and one by 6 points).
